# Pleomorphic Adenoma of the Palate: A Case Report

**DOI:** 10.7759/cureus.31003

**Published:** 2022-11-02

**Authors:** Zayd Berrerhdoche, Azzedine Lachkar, Drissia Benfadil, Fahd Elayoubi

**Affiliations:** 1 Department of Otolaryngology, Head and Neck Surgery, University Hospital Center Mohammed VI/Mohammed First University, Faculty of Medicine and Pharmacy, Oujda, MAR

**Keywords:** surgery, case report, treatment, palate, pleomorphic adenoma

## Abstract

A pleomorphic adenoma or mixed tumor is a heterogeneous benign tumor of the salivary glands. The most frequent site is the parotid gland. It is rare in the accessory salivary glands, preferably located in the oral mucosa (roof of the mouth, floor of the mouth, cheeks, and lips). The diagnosis of pleomorphic adenoma of the palate can only be made on a biopsy while remaining vigilant about the possible existence of other neoplastic foci within it. Definitive anatomopathology after excision is mandatory. Here, we present a clinical case of pleomorphic adenoma of the palate. We propose to specify the epidemiological, diagnostic, and therapeutic particularities of these tumors as well as their evolutionary characteristics.

## Introduction

The palatal vault is the site of a wide variety of tumors. Most are benign, some are true cancers, and others are difficult to classify and evaluate. The accessory salivary glands, spread throughout the upper aerodigestive tract, can be the site of tumors, mainly in the mucosa of the mouth (roof of the mouth, floor of the mouth, cheeks, lips), more rarely in the larynx or nasal cavity. The tumor varieties are numerous; the most common tumors that originate from the minor salivary gland are malignant tumors and benign tumors that recur in rare cases and can transform into carcinoma after a long evolution [1].

## Case presentation

**Figure 1:**
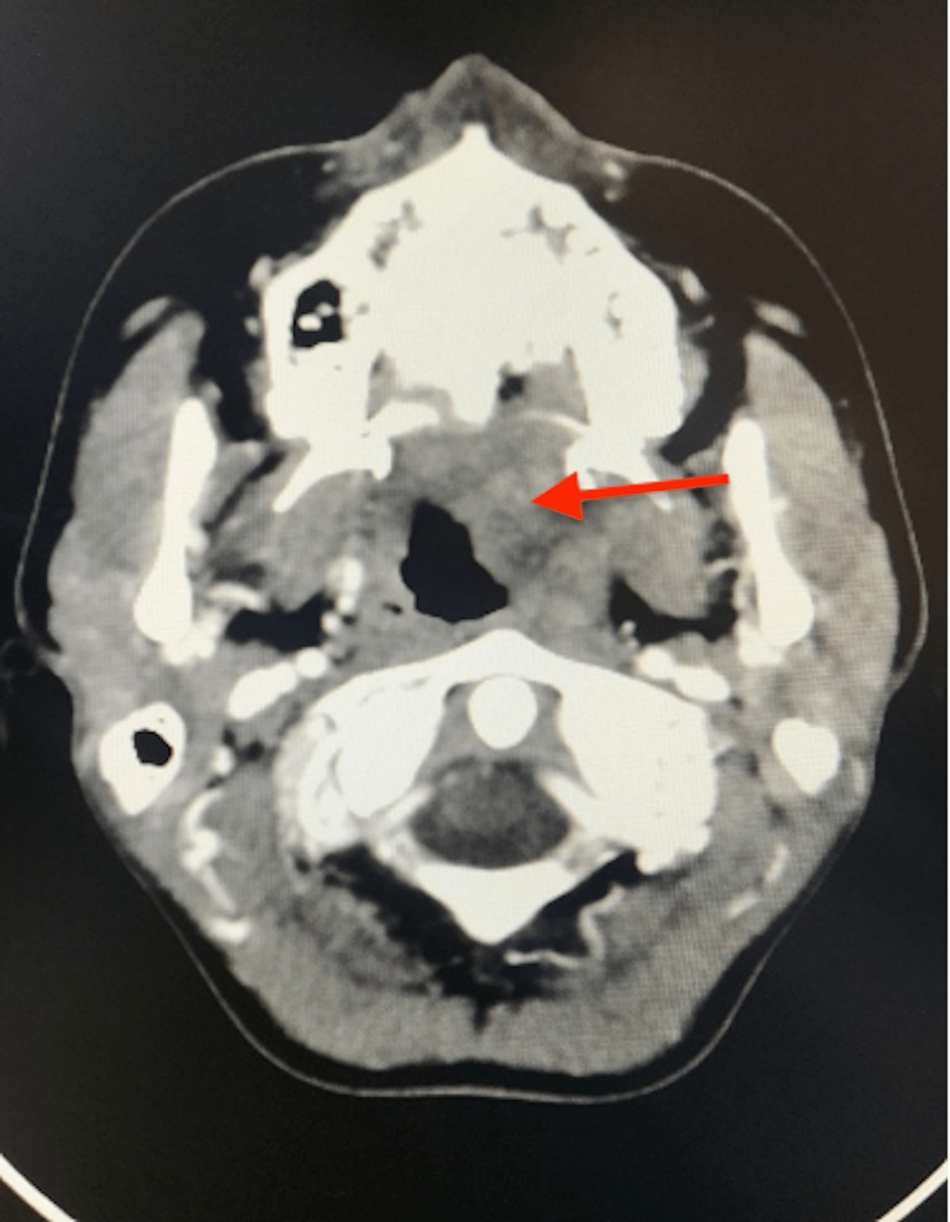
Contrast-enhanced computed tomography scan (axial section) showing a heterogeneous tissue lesion with moderately hypodense areas, well limited and responsible for a discrete mass effect on the oropharynx (red arrow). Presence of some subcentimeter bilateral jugulo-carotid nodes.

A 26-year-old female patient, with no specific pathological history, consulted in September 2021 for a swelling of the left soft palate. The swelling started three years earlier with the appearance of a small nodule that progressively increased in volume to fill the entire left hemipalate. Clinical examination revealed a swelling of the left hemipalate, 31 × 32 mm in diameter, firm, painless, not bleeding on contact, and lined by normal mucosa. There was no cervical adenopathy or other palpable cervicofacial mass. Computed tomography (CT) scan performed before and after injection of contrast medium showed a 32-mm mass of well-limited tissue appearance of the soft palate lateralized to the left but discreetly protruding from the median line of regular contours of non-univocal nature (Figure 1).

**Figure 2:**
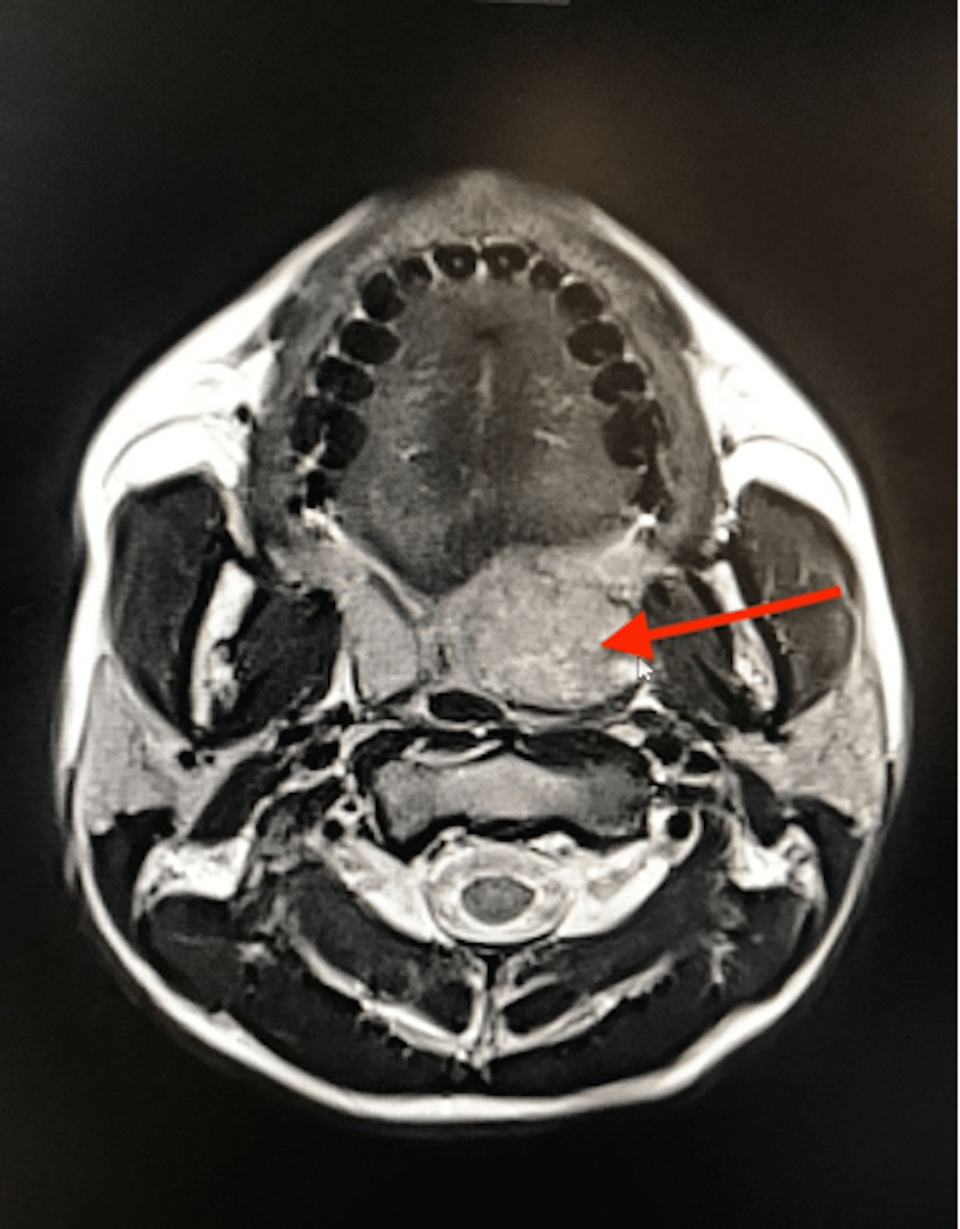
T1-weighted magnetic resonance imaging with contrast (axial section) showing a discrete heterogeneous contrast of the lesion (red arrow).

**Figure 3:**
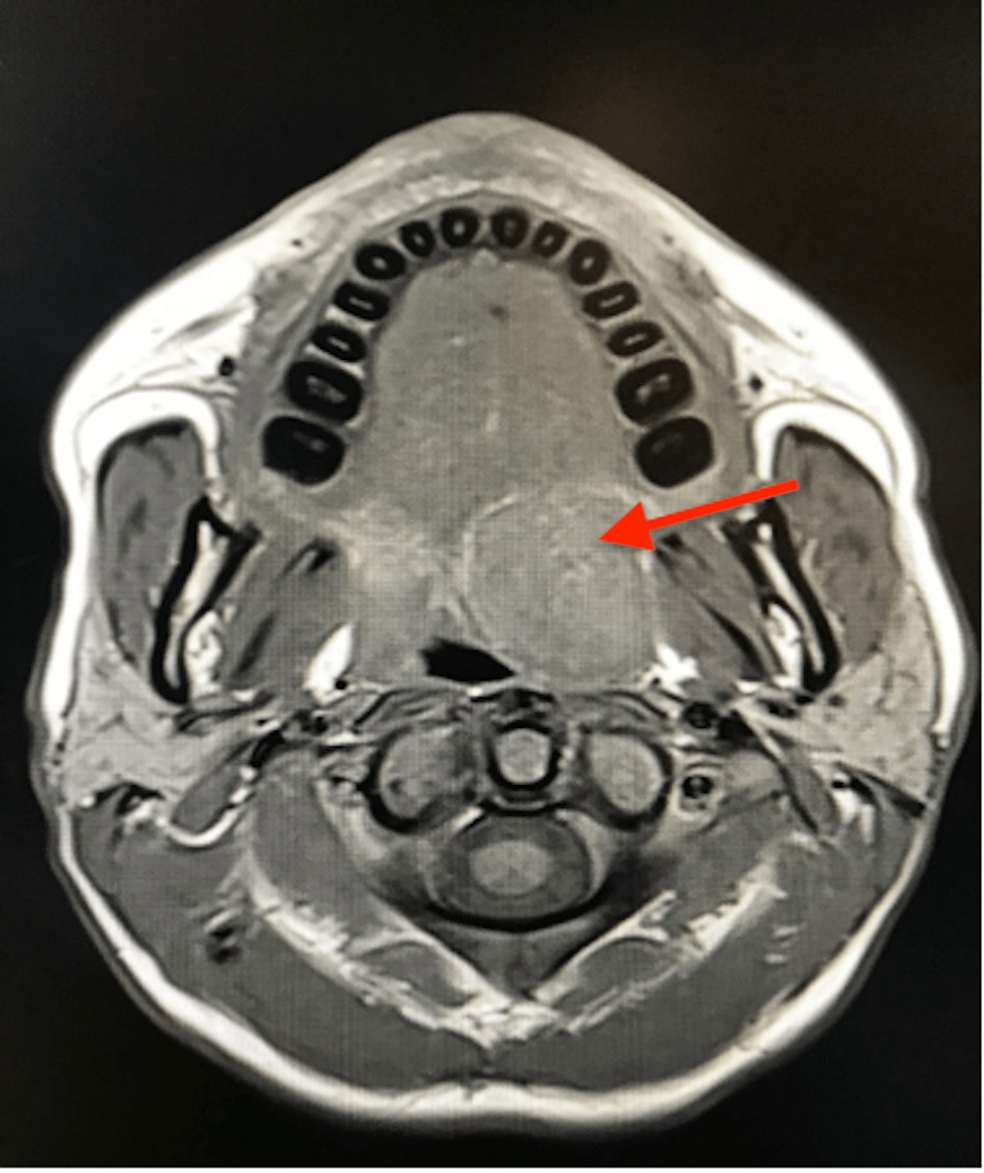
Magnetic resonance imaging in the T2 axial section showing a voluminous expansive process evaluated at 28 × 26 × 27 mm located at the junction of the soft palate and the left palatine tonsil presenting a low signal close to the signal of the tonsil but slightly more heterogeneous (red arrow).

Magnetic resonance imaging (MRI) showed a voluminous lesion appearing at the junction between the left soft palate and the palatine tonsil measuring 28 × 26 × 27 mm evoking a pleomorphic adenoma (Figures 2, 3). A biopsy was not performed.

**Figure 4:**
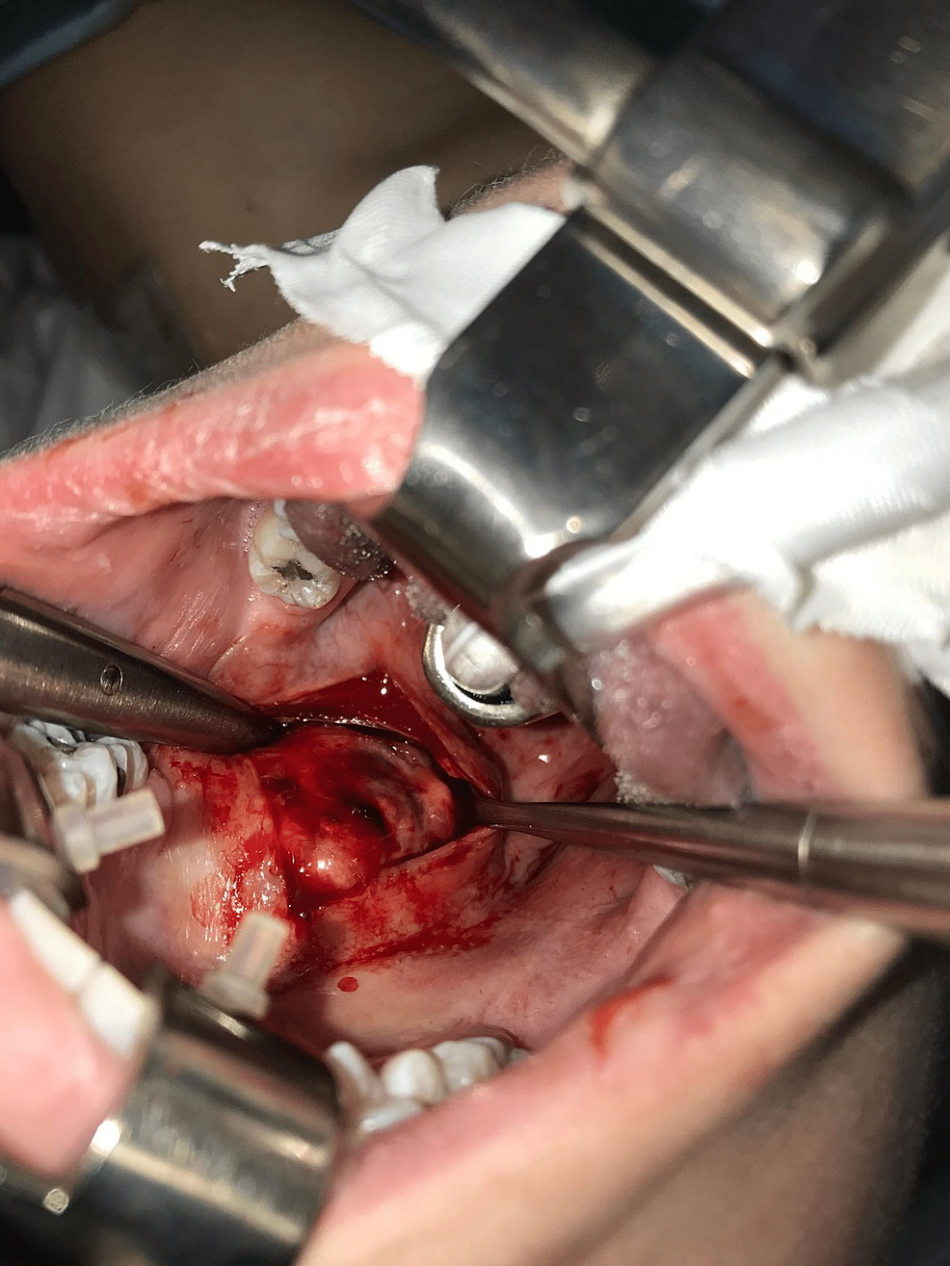
Intraoperative view of the pleomorphic adenoma.

The resection was considered total intraoperatively with a tight closure of the mucosa (Figure 4) after placing a mouth opener. The incision of the mucous membrane was made opposite to the tumor. The tumor was removed without difficulty and without any adhesion. Careful hemostasis was performed. The closure was done in two planes, namely, muscular and mucous.

**Figure 5:**
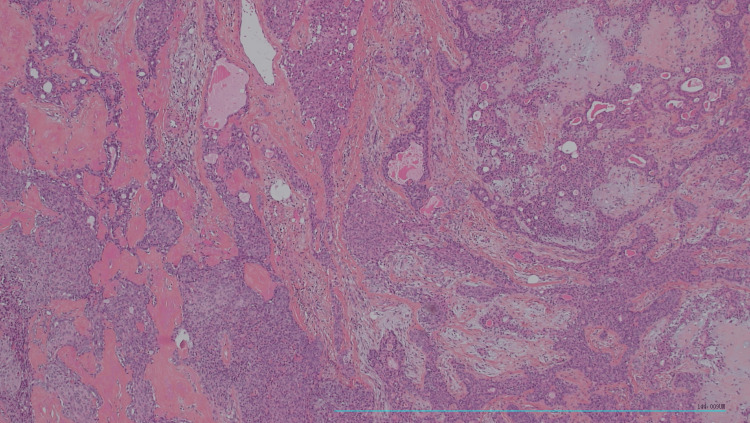
Histological examination of the surgical specimen showing an encapsulated tumor proliferation with a double contingent: a connective contingent made of spindle cells without cytonuclear atypia and an epithelial contingent made of tubes and trabeculae of cubic cells without cytonuclear atypia (hematoxylin and eosin, 100×).

The histological examination of the surgical specimen showed an encapsulated tumor proliferation with a double contingent: a connective contingent made of spindle cells without cytonuclear atypia and an epithelial contingent made of tubes and trabeculae of cubic cells without cytonuclear atypia (Figure 5).

We concluded that it was a pleomorphic adenoma. Feeding was provided for three days by a nasogastric tube. Postoperative care consisted of antibiotic therapy and local betadine care. Postoperative follow-up was simple. Control at 10 months of evolution did not reveal any local recurrence.

## Discussion

Tumors of the accessory salivary glands are very rare. The palate, with more than 50% of the accessory glands equally divided between hard and soft palate, represents the preferential site of accessory salivary tumors (two-thirds of cases). Pleomorphic adenoma of the roof of the mouth or soft palate occurs on average between the third and fifth decade of life and affects slightly more women than men [1,2].

Salivary tumors of the palate are characterized by discreet functional signs: slight discomfort when swallowing, and sensation of a foreign body. The palatal mucosa is respected for a long period only appearing blown by the tumor formation. They are generally firm and hard to palpation. At the exulceration stage, the tumor often appears grayish-white with necrotic areas [3]. Several tumor varieties can be encountered in the palate and pose a problem in the differential diagnosis of pleomorphic adenoma. In the context of benign tumors, it can be a monomorphic adenoma with basal or ductal cells. In malignant tumors, it may be a mucoepidermoid carcinoma, an adenoid cystic carcinoma (cylindroma), an acinar cell adenocarcinoma, or a low-grade polymorphic adenocarcinoma. Other lesions that may be seen are necrotizing sialometaplasia, mucocele, lymphoid hyperplasia, adenomatoid hyperplasia, primary lymphoma, and subacute necrotizing sialadenitis [4].

The definitive diagnosis is essentially based on the anatomical-pathological examination of the surgical specimen. Histologically, the pleomorphic adenoma presents as islands of spindle cells on a myxoid background, an internal layer of epithelial cells, and an external layer of myoepithelial cells. Three main subtypes have been identified, namely, myxoid (stroma accounts for 80%), cellular (with a predominance of myoepithelial cells), and mixed (classical type) [5]. The latter type is characterized by a mixture of polygonal epithelial cells and spindle-shaped myoepithelial cells on stroma that may be either mucoid, myxoid, cartilaginous, or hyaline. Areas of metaplasia may appear. In the oral cavitý, this tumor is unique in that it is not enveloped but surrounded by a fibrous pseudocapsule of variable thickness [6].

The treatment of pleomorphic adenoma of the palate is based on wide surgical excision to avoid the risk of recurrence [5,6].

## Conclusions

Tumors of the palate are rare and of various histological types. The possibility of a pleomorphic adenoma must be evoked in case of palate swelling. The literature confirms that this tumor is progressive and invasive in case of surgical abstention and it tends to recur locally and regionally. Complete removal is imperative. Regular locoregional surveillance after excision must be systematic for several years.
